# Biomarkers of muscle damage and oxidative stress and biomechanical responses of two different advanced footwear technology shoes to a 60-min running test in competitive long-distance runners

**DOI:** 10.3389/fphys.2025.1653896

**Published:** 2025-10-01

**Authors:** Alejandro Alda-Blanco, Fernando González-Mohíno, José María González-Ravé, Jordan Santos-Concejero

**Affiliations:** ^1^ Sports Training Laboratory, Faculty of Sport Sciences, University of Castilla-La Mancha, Toledo, Spain; ^2^ Department of Physical Education and Sport, University of the Basque Country UPV/EHU, Vitoria-Gasteiz, Spain

**Keywords:** inflammation, performance, cushioning, running shoes, marathon

## Abstract

**Objective:**

This study aimed to analyse the influence of Advanced Footwear Technology (AFT) on biomechanical, muscle damage, metabolic and oxidative stress markers in experienced long-distance runners.

**Methods:**

Using a counter-balanced randomized experimental design with twelve *tier 3* male runners, two AFTs were tested: *Cloudboom Strike* (CS) and *Cloudboom Echo* 3 (CE). All participants completed, in both conditions, a graded exercise test (GXT) to determine the VT_1_, followed by a prolonged effort test (60-min) at an intensity 10% above VT_1_. Finally a GXT to exhaustion to assess the changes in biomechanical parameters in a fatigued state was performed. 24-h before and after each visit, blood samples were drawn for muscle damage, metabolic and oxidative stress determination.

**Results:**

Creatin kinase (CK) increased post-visit in both CS and CE (*p =* 0.026; ES = 0.69 and *p =* 0.018; ES = 0.59; respectively). However, no other significant differences pre- and post-visit were found in lactate dehydrogenase (LDH), Interleukin-6 (IL-6), alanine aminotransferase (ALT), cortisol or total antioxidant status (TAS) in either the CS or CE conditions. There were no differences between conditions in any of the biomarkers measured, although participants perceived lower DOMS post-24 h with the CS model (*p* = 0.016; ES = 0.71). We found no shoe × time interaction in any biomechanical parameter evaluated during the prolonged effort or any variable at any speed stage between footwear conditions in the GXT to exhaustion.

**Conclusion:**

According to our results, both the *Cloudboom Strike* and the *Cloudboom Echo* 3 models appear to provide minimal biomarker responses under these conditions after a 60-min treadmill run, regards to reducing muscle damage, oxidative and metabolic stress, soreness, and inflammation. This indicates attenuated biomarker responses when compared to previous studies with traditional footwear.

## 1 Introduction

Long-distance running performance is largely reliant on a complex interaction of physiological factors, including maximal oxygen uptake (VO_2_max), lactate threshold and running economy, as well as biomechanical parameters such as stride length and frequency, ground contact time or vertical oscillation, which contribute to optimized force production and reduced energy cost, supporting a more economical and faster running (Joyner, 1991; Santos-Concejero et al., 2014).

During running, there is a substantial eccentric component (Black et al., 2022), which occurs when the force applied to the muscle exceeds the tension produced by the muscle itself, resulting in the lengthening of the musculotendinous system (Hody et al., 2019; Bontemps et al., 2020). This eccentric component has been reported to induce high mechanical strain, resulting in moderate to severe exercise-induced muscle damage (Black et al., 2022; Bontemps et al., 2020; Millet and Lepers, 2004), that triggers acute biomechanical changes such as an increased stride frequency and decreased stride length (Kim et al., 2018). Similarly, there is evidence that prolonged strenuous exercise induces metabolic stress (Knechtle and Nikolaidis, 2018; Sadowska-Krępa et al., 2021) and promotes the production of reactive oxygen species (ROS) in response to the systemic inflammation increment caused by muscle damage (Powers et al., 2016).

Previous research has reported increases in the concentration of creatine kinase (CK), lactate dehydrogenase (LDH), transaminases, pro-inflammatory and anti-inflammatory cytokines such as the interleukin-6 (IL-6), chemokines, and associated inflammatory mediators after strenuous endurance exercise (Sugama et al., 2012; Suzuki et al., 2003; Tominaga et al., 2021; Lecina et al., 2022). All this, ultimately, leads to substantial muscle soreness, inflammation and swelling, and the loss of muscle function, which can persist for several days (Black et al., 2022; Kyröläinen et al., 2000). Since muscle damage in runners results in greater performance impairments than other exercises with little or no impact (Black et al., 2022; Millet and Lepers, 2004), it has been suggested that it may even be the main performance constraint in ultra-endurance running events (Tiller and Millet, 2025).

In that regard, advanced footwear technology (AFT) development in the last few years has been a revolution (Rodrigo-Carranza et al., 2022a). AFT shoes are characterised by the presence of a curved carbon fiber plate aimed at optimising longitudinal bending stiffness (Rodrigo-Carranza et al., 2022b; Rodrigo-Carranza et al., 2024) embedded in lightweight, resilient midsole foams that enhance cushioning and mechanical energy return (Rodrigo-Carranza et al., 2022b; Hoogkamer et al., 2018; Winn et al., 2025). Numerous studies have reported that AFT can improve running economy and performance (Rodrigo-Carranza et al., 2022a; Rodrigo-Carranza et al., 2022b; Rodrigo-Carranza et al., 2024; Hoogkamer et al., 2018), and it may also reduce fatigue, muscle damage and soreness in recreational runners after a marathon (Kirby et al., 2019). The question arises whether AFT also provides a small biomarker response for muscle damage and metabolic and oxidative stress during long-distance running in experienced runners.

Therefore, in this study we aimed to analyse the influence of two different AFT-which differ in their mechanical properties-on biomechanical, neuromuscular, muscle damage, metabolic and oxidative stress markers in experienced runners during a strenuous long-distance running bout. We hypothesized that the difference in mechanical characteristics, such as energy return or resistance, of these shoe models would elicit differential biochemical and biomarker responses in experienced runners.

## 2 Materials and methods

### 2.1 Participants

Twelve highly trained/national level -*tier 3-* male runners (McKay et al., 2022) (age: 30.83 ± 8.36 years; body mass: 65.98 ± 7.72 kg; height: 174.5 ± 4.75 cm; performance: WA score: 820.83 ± 107.34 points; weekly kms in the last 6 months: 84.16 ± 15.78) were recruited for the study. Based on a previous study with similar methodology (Jürgenson et al., 2021), *post hoc* sample size calculation was performed using the reported effect size for CK (Cohen’s d = 1.5–1.6) to estimate statistical power. Using a paired-samples framework (equivalent to the Shoe × Time interaction in our design, α = 0.05, two-tailed), sample size of n = 12 yields *post hoc* power >99% for CK (0.997–0.999).

The inclusion criteria were the following: participation in endurance training for at least 5 days per week during the previous 6 months without injury, >650 world athletic score in 10 km to marathon events, wearing a men’s EU 40–45 shoe size, and to be experienced AFT users.

Prior to the study, all participants were informed about the testing protocols and possible risks involved and provided written informed consent. The study was performed in accordance with the principles of the Declaration of Helsinki (Worl d-Medical-Association, 2025) and the experimental protocols were approved by the Ethics Committee of the local university (CEIm2176).

### 2.2 Experimental design

We tested the effects of two different AFT with varying compliance and resilience properties on the durability of biomechanical and neuromuscular parameters, as well as on muscle damage, metabolic and oxidative stress using a counter-balanced randomized experimental design. The experimental design consisted of two visits on different days within 7–10 days in between to ensure full recovery. All participants refrained from strenuous exercise in the previous 48 h. Participants were asked to avoid caffeine and alcohol intake within 24 h before the visit. In addition, during the second session, participants were instructed to follow the same dietary, hydration, and sleep routines as in the first session. The laboratory sessions were carried out in the same laboratory with similar environmental conditions for both footwear conditions (529 m altitude; 21.4 ± 1.2 vs. 20.8 °C ± 1.2 °C [*p* = 0.10], and 44.2% ± 9.2% vs. 44.1% ± 12.6% [*p* = 0.49] relative humidity). Twenty-four hours before and after each visit, blood samples were collected to determine changes in muscle damage, metabolic, and oxidative stress markers.

### 2.3 Footwear characteristics

**FIGURE 1:**
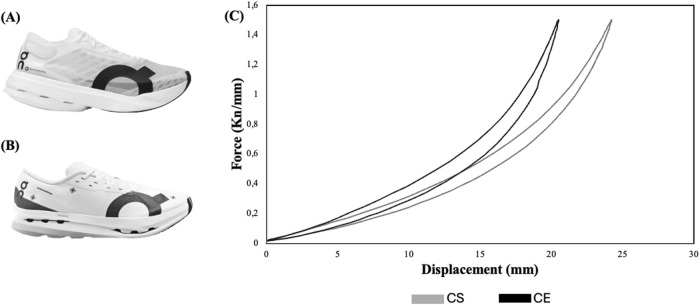
Shoe conditions: **(A)** *Cloudboom Strike* (CS); **(B)** *Cloudboom Echo 3* (CE); **(C)** Force-displacement representation for AFT conditions.

Two AFTs were tested: *Cloudboom Strike* (CS) (ON Holding AG, Switzerland) and *Cloudboom Echo* 3 (CE) (ON Holding AG, Switzerland) (Figure 1). The mechanical characteristics are shown in Table 1. The main common aspects were that both AFT models have PEBA foam and a carbon plate to increase longitudinal bending stiffness. According to Table 1, the CE is heavier (219 g vs 204 g), has lower stack height (37.0 mm vs 39.5 mm), less deformation (20.22 mm vs 24.15 mm), higher stiffness (73.29 N/mm vs 61.41 N/mm), but lower energy return (8.27 J vs 10.46 J) compared to the CS. These mechanical differences suggest that the CE may provide a stiffer platform, while the CS, with greater deformation and energy return, may offer more cushioning and attenuate impact forces, potentially reducing muscle damage or fatigue. The researchers fitted the participants with the shoe size for all sessions, and the participants were not allowed to manipulate the footwear at any time during the testing protocol. While the investigators were aware of the footwear characteristics, this information was not disclosed to the participants. To standardise warm-up and determine the first ventilatory threshold (VT_1_) without the influence of both experimental AFT, the same control shoe model was used in both sessions (*Cloudboom Zone* [CZ] (ON Holding AG, Switzerland)). The CZ was selected because, although it shares PEBA foam construction with the experimental AFT, it lacks the curved carbon plate that characterize CS and CE. Therefore, it provides a neutral baseline that allows assessment of the participants’ physiological responses without the enhanced propulsion of the experimental shoes, minimizing potential bias in VT_1_ determination and warm-up performance differences between shoe conditions.

**TABLE 1:** Characteristics of experimental AFT for EU42.

Characteristics	Cloudboom Strike	Cloudboom Echo 3
Weight (g)	204	219
Stack height (mm)	39.5	37.0
Deformation (mm)	24.15	20.22
Stiffness (N/mm)	61.41	73.29
Stored Energy (J)	12.23	10.03
Energy loss (J)	1.76	1.76
Energy return (J)	10.46	8.27
Resistance (%)	85.57	82.43

The force-displacement relationship of the AFT tested was determined by a compression test on the complete shoe (including the upper) where a custom-made structure simulating a size EU42 foot was attached to a material testing machine that measures force and displacement (Zwick/Roell, Ulm-Einsingen, Germany) (Figure 1). A force of 1800 N was applied at 2 Hz in the forefoot during 60 cycles, and the average of the last 10 cycles was used to characterize both AFTs. Longitudinal bending stiffness was calculated as the slope between 0 and 1500 N and energy return as the area of hysteresis loop. Also, resistance was calculated as the percentage of stored mechanical energy that is returned during shoe deformation–recovery, representing the efficiency of the midsole in minimizing energy loss and maximizing energy return.

### 2.4 Procedure

Participants visited the laboratory twice, one per shoe condition. In each visit, participants carried out the same protocol. A graded exercise test (GXT) was performed to determine the VT_1_, followed by a prolonged effort (60-min) test at an intensity 10% above VT_1_, and finally a GXT to exhaustion to assess the changes in biomechanical parameters in a fatigued state. Each test was separated by a 5-min rest. All tests were performed in the same treadmill (HP Cosmos Pulsar, Germany) with a constant slope of 1% to reflect the energetic cost of outdoor running (Jones and Doust, 1996) and a belt width of 50 cm.

First, as a warm-up for the durability test and to standardise the speed for both shoe conditions, participants performed a GXT to determine VT_1_. The test started at 10 km·h^-1^ for a 5-min warm-up and then, the speed was increased to 12 km·h^-1^ for a minute and finally it was increased again by 1 km·h^-1^ every minute until the VT_1_ speed was obtained. The VT_1_ was assessed using a mixed method following previous recommendations (Jones and Carter, 2004; Keir et al., 2022) based on the visual inspection of carbon dioxide production (VCO_2_) and minimum value of VE/VO_2_. This inspection was performed by two researchers. Gas analysis was performed using the CPX Ultima Series MedGraphics (St. Paul, MI, United States), which was calibrated before each session (CO_2_ 4.10%; O_2_ 15.92%). The zirconia O_2_ analyzer has a response time of <0.80 ms and accuracy of ±0.03%, and the CO_2_ analyzer has a response time of <130 ms and accuracy of ±0.10%. In the second day, the GXT was repeated up to the same VT_1_ speed as in the first day to standardize pre-test fatigue.

To standardise the speed of the prolonged effort for all the participants (same relative intensity), the running speed was set 10% above VT_1_. The duration of the prolonged effort was 60 min. Throughout the test, biomechanical variables were continuously monitored. To evaluate how the athletes responded during the test, the hour was divided into 10-min intervals, and the average measurements from the last 5 min of each interval were used for comparison across the different time points. Participants performed the test at a mean speed of 15.63 ± 0.95 km·h^-1^.

### 2.5 Measures

#### 2.5.1 Muscle damage blood markers measures

Five mL venous blood samples were drawn from the participants’ antecubital veins into 5-mL lithium-heparin tubes (Vacutainer; Becton-Dickinson, Franklin Lakes, NJ) 24 h before and after each visit. Plasma was separated by centrifugation for 8 min at 4,000 rpm at 4 °C (RotoFix 32A, Hettich, Germany) and stored at −80 °C until analysis at *Abacid Innovation Human Labs*. Serum levels of CK, alanine aminotransferase (ALT), lactate dehydrogenase (LDH), interleukin-6 (IL-6), total antioxidant status (TAS) and cortisol levels were measured as key indicators of muscle damage, oxidative and metabolic stress (Brancaccio et al., 2010; Finsterer and Drory, 2016; Thirupathi et al., 2020). Serum CK, LDH and ALT activities were measured spectrophotometrically using the Roche Cobas 6,000 c501 automated analyser (Roche Diagnostics, Switzerland). The enzyme activities were expressed as U·L^-1^ where 1U of activity corresponds to 0.0167 μkat⋅L^-1^. Serum IL-6 and cortisol levels were estimated by electrochemiluminescence immunoassays (ECLIA) from Roche Diagnostics (Elecsys® IL-6 and Elecsys® Cortisol II, respectively) on Cobas analysers. TAS values were measured by spectrophotometric methods (Randox Laboratories Ltd., Crumlin, United Kingdom). All biochemical tests were performed as per PN-EN ISO 9001:2015 and the test manufacturers’ instructions by a certified laboratory.

#### 2.5.2 Biomechanics measures

The main spatiotemporal parameters of the gait cycle (contact time [CT], step frequency [SF], leg stiffness [Kleg] and vertical oscillation [VO]) were measured for each step during three tests using an inertial measurement unit (IMU) (Stryd Power Meter, Stryd Inc. Boulder CO, United States) with a sampling frequency of 1,000 Hz. A tri-axial trunk accelerometer (TTA) (RunEASI NV, Tienen-Flemish-Brabant, Belgium) was also used to assess dynamic stability (DS), impact duration (ID), and impact magnitude (IM). The TTA was positioned on the participants’ lower back at the level of the L3 vertebra using an elastic belt and the IMU was attached to the dorsal aspect of the shoe using the manufacturer-provided holder.

DS was defined as the side-to-side movement of the pelvis during the landing phase expressed as proportion between both legs. ID as the time it takes for the body to absorb the impact force after foot contact (ms), and IM as the peak vertical impact force experienced during each footstep (G force). Both the IMU device (Rodriguez-Barbero et al., 2024) and TTA (Moe-Nilssen and Helbostad, 2004) have shown adequate validity and reliability compared to optical measurement devices and slow-motion recording to measure spatiotemporal parameters and Kleg (Imbac et al., 2020). In addition, step length (SL) was calculated from speed, CT, and SF (García-Pinillos et al., 2021). Flight time (FT) was derived from CT and SF (García-Pinillos et al., 2021), and step time (ST) was determined using CT and FT. Finally, duty factor (DF) was calculated as the percentage of CT in stride time (CT/(2·ST)·100) (van Oeveren et al., 2021).

#### 2.5.3 Subjective measures

Delayed onset muscle soreness (DOMS) was measured using the seven-point Likert scale (Connolly et al., 2003). Participants were asked to rate the overall level of DOMS felt in both legs during 24 h post each test according to the following verbal anchors: 0 a complete absence of soreness; 1 a light pain felt only when touched/a vague ache; 2 a moderate pain felt only when touched/a slight persistent pain; 3 a light pain when walking up or down stairs; 4 a light pain when walking on a flat surface/painful; 5 a moderate pain, stiffness, or weakness when walking/very painful; 6 a severe pain that limits my ability to move.

### 2.6 Statistics

All data are presented as means ± SD. The statistical analyses of data were performed using the Statistical Package for the Social Sciences 21.0 software package (SPSS Inc., Chicago, IL, United States). Data were screened for normality of distribution and homogeneity of variances using a Shapiro-Wilk normality test and a Levene test, then Q-Q plots were examined to confirm distributional assumptions. Two-way analysis of variance (ANOVA) with repeated measures (shoe condition*time) was performed to assess biomechanical differences across the 60-min test and paired *t*-test to evaluate differences for the same variables at the GXT. When significant differences were found, multiple pairwise comparisons with Bonferroni adjustment were made. Also, a paired Student’s t-test for the comparison of muscle damage, oxidative and metabolic stress biomarkers between conditions. Effect sizes (ES) were calculated using Cohen’s *d* (Cohen, 1988) and interpreted as trivial (<0.2) small (≥0.2 and <0.6), moderate (≥0.6 and <1.2) and large (≥1.2 and <2) according to the scale proposed by Hopkins et al. (Hopkins et al., 2009). Significance for all analyses was set at *p* < 0.05.

## 3 Results

### 3.1 Muscle damage, oxidative and metabolic stress biomarkers

**FIGURE 2:**
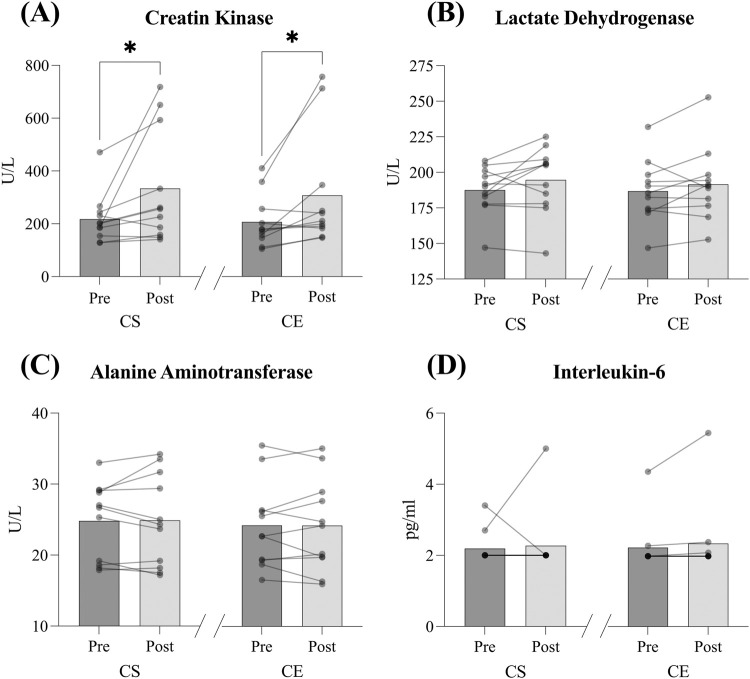
Muscle damage biomarkers -creatin kinase **(A)**, lactate dehydrogenase **(B)**, Interleukin-6 **(C)** and alanine aminotransferase **(D)**- pre- and post-visit in the *Cloudboom Strike* (CS) and *Cloudboom Echo 3* (CE) footwear conditions. CK significantly increased post-visit in both conditions (moderate effects). No significant pre-to post-visit changes were observed in LDH (small effects), IL-6 (trivial effects), or ALT (trivial effects). *Differences between pre- and post-visit, p < 0.05.

Figure 2 and Table 2 presents muscle damage biomarkers pre- and post-visit in each of the experimental conditions. CK increased post-visit in both CS and CE (*p =* 0.026; ES = 0.69 and *p =* 0.018; ES = 0.59 respectively, Figure 2A). However, no other significant differences pre- and post-visit were found in LDH (ES = 0.35 and ES = 0.20, *small effect*, Figure 2B), IL-6 (ES = 0.11 and ES = 0.13, *trivial effect*, Figure 2C) or ALT (ES = 0.013 and ES = 0.002*, trivial effect,* Figure 2D) in either the CS or CE conditions.

**TABLE 2:** Biomarkers of muscle damage and oxidative and metabolic stress 24-h before and after the running test.

	Pre CS		Post CS		Pre CE		Post CE	
	Mean	SD	Mean	SD	Mean	SD	Mean	SD
Muscle damage								
CK (U/L)	217,89	94,81	333,75	214,89	206,87	97,71	307,95	218,33
LDH (U/L)	187,52	17,03	194,74	23,49	187,45	22,18	192,33	26,01
IL-6 (pg/mL)	2,19	0,45	2,27	0,90	2,25	0,72	2,36	1,05
ALT (U/L)	24,82	5,39	24,90	6,48	24,35	6,10	24,34	6,60
Oxidative and Metabolic Stress								
Cortisol (ug/dL)	17,35	3,92	15,66	3,11	16,53	3,16	16,47	2,87
TAS (mmol/L)	1,62	0,08	1,67	0,11	1,62	0,08	1,63	0,12

CS, cloudboom strike; CE, Cloudboom Echo 3; SD, standard deviation; CK, creatin kinase; ALT, alanine aminotransferase; LDH, lactate dehydrogenase; IL-6, interleukin-6; TAS, total antioxidant status.

**FIGURE 3:**
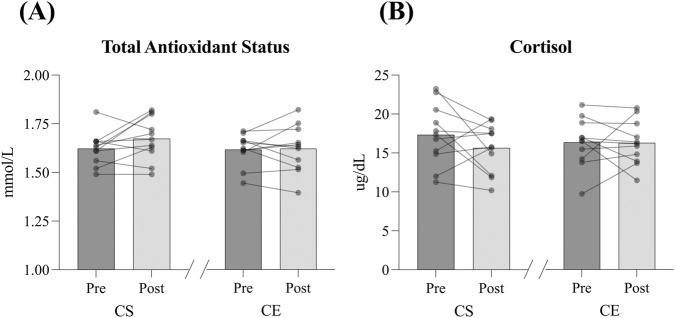
Oxidative and metabolic stress biomarkers–total antioxidant status **(A)** and cortisol **(B)**- pre- and post-visit in the *Cloudboom Strike* (CS) and *Cloudboom Echo 3* (CE) footwear conditions. No significant pre-to post-visit differences were observed in either condition. Effect sizes were trivial-to-moderate.

Oxidative and metabolic stress biomarkers pre- and post-visit are shown in Figure 3 and Table 2. We found no differences in TAS (ES = 0.51 and ES = 0.05 for CS and CE, respectively, *trivial* and *small effect*, Figure 3A) or cortisol (ES = 0.047 and ES = 0.02 for CS and CE, respectively, *trivial effect,* Figure 3B) pre- and post-visit in either of the experimental conditions. Although these results did not reach significance, the small effect size for TAS in the CS condition suggests a potential trend toward altered antioxidant status.

**FIGURE 4:**
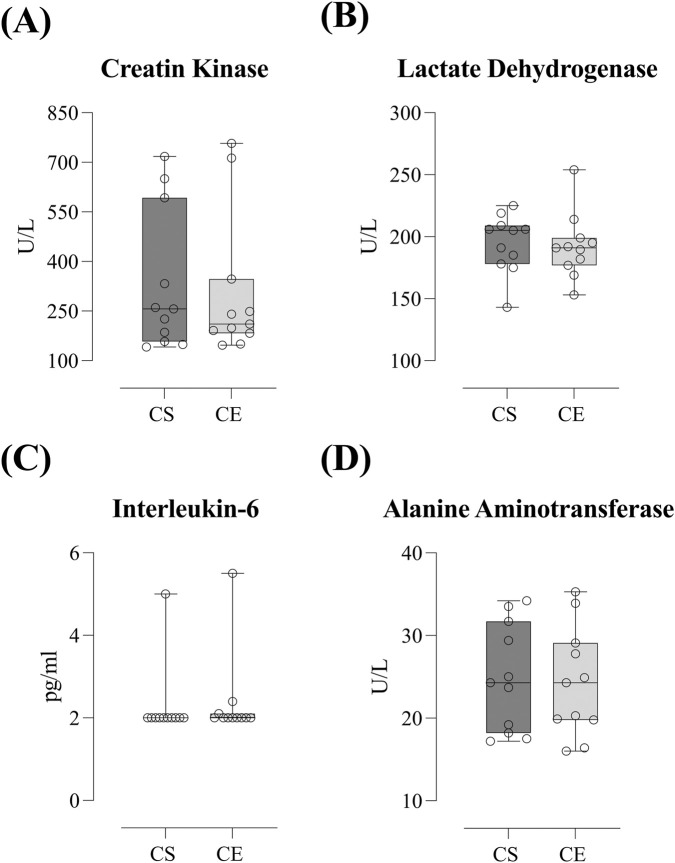
Muscle damage biomarkers comparison -creatin kinase **(A)**, lactate dehydrogenase **(B)**, Interleukin-6 **(C)** and alanine aminotransferase **(D)**- between *Cloudboom Strike* (CS) and *Cloudboom Echo 3* (CE) footwear conditions. No significant differences were found in post-visit values between CS and CE (trivial effects). However, LDH showed a significantly greater relative increment in CS compared to CE (moderate effect).

Figure 4 depicts muscle damage biomarkers’ comparison between CS and CE. There were no differences in either relative increments or absolute post-visit CK (333.75 ± 214.89 vs. 307.94 ± 218.33 U·L^-1^ for CS and CE, respectively; ES = 0.11, *trivial effect,* Figure 4A), LDH (194.73 ± 23.49 vs. 192.32 ± 26.01 U·L^-1^ for CS and CE, respectively; ES = 0.09, *trivial effect,* Figure 4B), IL-6 (2.72 ± 0.90 vs. 2.36 ± 1.04 pg·mL^-1^ for CS and CE, respectively; ES = 0.09, *trivial effect,* Figure 4C), or ALT (24.90 ± 6.48 vs. 24.33 ± 6.60 U·L^-1^ for CS and CE, respectively; ES = 0.08, *trivial effect,* Figure 4D) between conditions. However, we found that, when analysing the relative increment from baseline, LDH increased to a greater extent in the CS condition when compared to CE (*p* = 0.030, ES = 0.93, *moderate effect*). In addition, participants perceived a lower DOMS post-24 h of the protocol with the CS shoe model when compared to CE (mean ± SD: 2.0 ± 1.47 vs. 2.79 ± 1.34; median [IQR]: 2.0 [IQR: 0.75–3.0] vs 3.0 [IQR: 2.0–3.0] in Likert scale points; *p* = 0.016; ES = 0.71, *moderate effect*).

**FIGURE 5:**
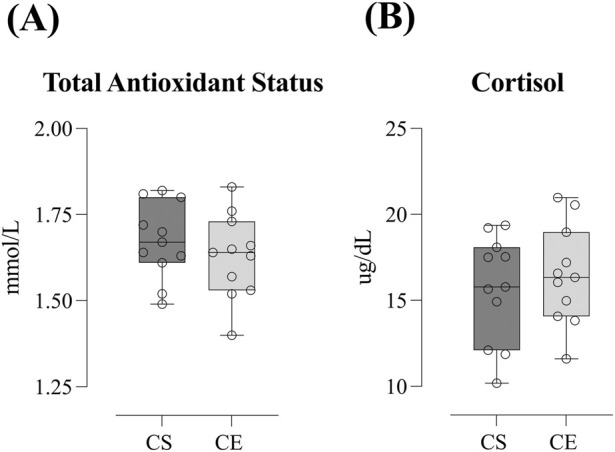
Oxidative and metabolic stress biomarkers comparison–total antioxidant status **(A)** and cortisol **(B)**- between *Cloudboom Strike* (CS) and *Cloudboom Echo 3* (CE) footwear conditions. No significant differences were observed between CS and CE in either absolute post-visit values or relative increments. Effect sizes indicated small differences.

Lastly, oxidative and metabolic stress biomarkers’ comparison between conditions is depicted in Figure 5. We saw no differences in either relative increments or absolute post-visit TAS (1.67 ± 0.11 vs. 1.62 ± 0.12 mmol·L^-1^ for CS and CE, respectively; ES = 0.38, *small effect,* Figure 5A) or cortisol (17.34 ± 3.91 vs. 16.46 ± 2.87 μg·dL^-1^ for CS and CE, respectively; ES = 0.25, *small effect,* Figure 5B) between conditions.

### 3.2 Biomechanical variables

**FIGURE 6:**
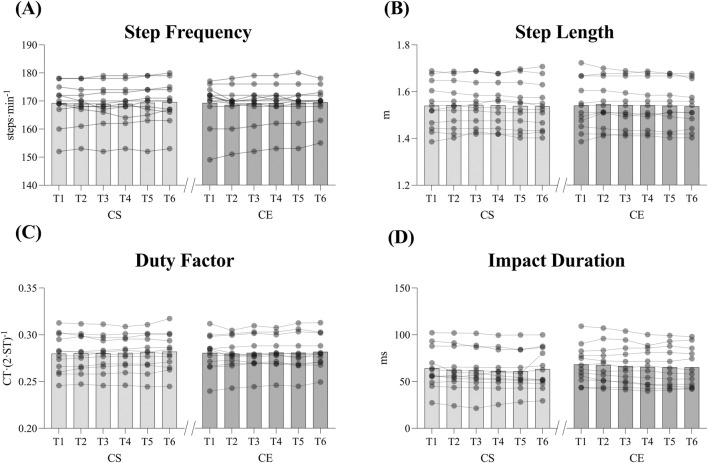
Biomechanical changes across prolonged effort test in Step Frequency **(A)**, Step Length **(B)**, Duty Factor **(C)** and Impact Duration **(D)**. Bars represent mean values and lines individual data. Over time, SF and DF increased significantly, while SL, and ID decreased significantly. No pairwise differences were detected between footwear conditions at any time point.

There was no significant shoe × time interaction in any biomechanical parameter evaluated during the prolonged effort, indicating that temporal changes occurred similarly in both footwear conditions. For the time factor, significant differences were found for several parameters measured with IMU. As shown in Figure 6, SF and DF increased across time points (*p* = 0.02, ηp2 = 0.805 and *p* = 0.008, ηp2 = 0.854, respectively). Also, VO, SL and FT decreased during the test (*p* = 0.04, ηp2 = 0.757; *p* = 0.022, ηp2 = 0.799 and *p* = 0.003, ηp2 = 0.891 respectively). When measuring with a TTA, ID decreased as well (*p* = 0.011; ηp2 = 0.837). Although significant differences were found for time in the variables previously described, no differences were found for any variable in the pairwise comparisons. Biomechanical variables of the incremental test were analysed for speeds between 12 km·h^-1^ and 20 km·h^-1^, as all participants performed at least 30 s of the last 20 km·h^-1^ stage. No other differences were found for any variable at any speed stage between footwear conditions.

## 4 Discussion

The main finding of this study was that both AFT shoes tested, the *Cloudboom Strike* and the *Cloudboom Echo* 3 models, resulted in minimum muscle damage, metabolic and oxidative stress, as the only muscle damage biomarker that increased post-exercise was the CK (Figure 2), with no changes in other muscle damage (LDH and ALT), inflammation (IL-6), metabolic (cortisol) or oxidative stress biomarkers (TAS) (Figures 2, 3). This contrasts with previous research reporting that strenuous long-distance running can lead to significant LDH (Jürgenson et al., 2021), ALT (Jastrzębski et al., 2015), IL-6 and cortisol (Vezzoli et al., 2016) and oxidative stress increases (Vezzoli et al., 2016). Therefore, according to our results, both AFT models appear to provide notable minimal biomarker response after a 60-min treadmill run with regards to reducing muscle damage, oxidative and metabolic stress, soreness, and inflammation, which may indicate an attenuated biomarker response when compared to previous studies of traditional footwear.

It is well-known that strenuous prolonged running results in moderate to dramatic increments in muscle damage biomarkers post-exercise (Brancaccio et al., 2010; Miles et al., 2008), and the activity of enzymes such as the CK, LDH or ALT is commonly used in that regard (Noakes et al., 1983; Kim et al., 2007; Kim et al., 2009). Previous research has reported changes as high as 10-fold–20-fold increases in CK activity 24-h post strenuous long-distance running (Jastrzębski et al., 2015; Kłapcińska et al., 2013), which contrasts with the relatively small <2-fold increase observed in this study (Figure 2). Similarly, the literature reports four-fold increases in ALT activity and significant LDH increases after long-distance running (Lippi et al., 2011), indicative of considerable damage to the skeletal muscles, whereas we found no differences pre- and post-exercise in ALT or LDH values when running in either of the AFT shoe conditions (Figure 2).

Strenuous exercise has also been reported to induce oxidative stress through enhanced formation of ROS (Leeuwenburgh and Heinecke, 2001). Previous studies have found increased oxidative stress in long-distance runners (Briviba et al., 2005; Liu et al., 1999; Vuorimaa et al., 1999), and ironman competitors (Reichhold et al., 2009) which contrasts with our findings, as we found no differences in TAS pre-post in either the *Cloudboom Strike* and the *Cloudboom Echo* 3 shoe models after the prolonged effort test (Figure 3). Oxidative stress is suggested to induce inflammation by activating different transcription factors, resulting in the expression of inflammatory pathway genes (Hussain et al., 2016). Therefore, the apparently attenuated post-exercise oxidative stress observed in this study may partially explain the unchanged pro-inflammatory cytokine IL-6 measurements after the prolonged effort test in the CS and CE shoe conditions (Figure 2).

In that sense, one possible explanation could be the fact that in the previous literature, the footwear models were not considered AFT as in our study, and the characteristics attributed (resilience and energy return) could impact the muscle damage biomarkers. However, in our study, a control condition (non-AFT) was not included; therefore, methodological differences between studies—such as exercise duration, blood sampling intervals, and the training status of participants—may partly account for the smaller biomarker responses observed here. Accordingly, while our findings suggest that both AFT models are associated with attenuated biomarker changes after a 60-min treadmill run, these differences might not be attributed exclusively to footwear effects but also considered within the context of protocol design and participant characteristics.

Increased delayed onset muscle soreness (DOMS), muscle damage and inflammation have been observed during intense and/or long-distance running as well (Kirby et al., 2019). All this leads to acute to chronic loss of muscle function, (Black et al., 2022; Kyröläinen et al., 2000), which is, ultimately, associated with running performance impairments (Black et al., 2022; Millet and Lepers, 2004; Tiller and Millet, 2025). The fact that both CS and CE conditions in this study resulted in relatively small muscle soreness post-exercise (mean ± SD: 2.0 ± 1.47 vs. 2.79 ± 1.34; median [IQR]: 2.0 [IQR: 0.75–3.0] vs 3.0 [IQR: 2.0–3.0] in Likert scale points) is, therefore, in agreement with evidence indicating that softer surfaces -as the ones of CS and CE due to their high cushioning-may lessen the DOMS response (Brown et al., 2017; Skinner et al., 2015). Our results are also in line with Kirby et al., (Kirby et al., 2019), who found that after a marathon, post-race indicators of muscle damage and associated inflammation were lower (LDH<15% and IL-6 <43%), in runners wearing AFT shoes (*Nike Vaporfly 4%)* when compared to traditional shoes (*Nike Zoom Pegasus*). They also reported that AFT resulted in less overall fatigue and less soreness (<22% and <46%).

Our results and the aforementioned studies clearly suggest that AFT shoes confer significant benefit effects against exercise-induced muscle damage, oxidative stress and inflammation. However, this is the first study comparing how different longitudinal bending stiffness -one of the key aspects of AFT shoes when compared to traditional ones-affects such responses. The *Cloudboom Echo* 3 (CE) is a stiffer (73.29 vs. 61.41 N mm^-1^) and less resistant to deformation (24.15 vs. 20.22 mm) model, whereas the *Cloudboom Strike* (CS) stores more energy (12.23 vs. 10.03 J) and displays a higher energy return (10.46 vs. 8.27 J) (Table 1).

Interestingly, participants reported lower DOMS 24 h post-exercise with the CS shoe model when compared to CE (*p* = 0.016; ES = 0.71, *moderate effect*). However, since we found no differences either relative increments or absolute post-visit CK (333.75 ± 214.89 vs. 307.94 ± 218.33 U·L^-1^), LDH (194.73 ± 23.49 vs. 192.32 ± 26.01 U·L^-1^), IL-6 (2.72 ± 0.90 vs. 2.36 ± 1.04 pg mL^-1^), ALT (24.90 ± 6.48 vs. 24.33 ± 6.60 U·L^-1^), cortisol (17.34 ± 3.91 vs. 16.46 ± 2.87 μg·dL^-1^) or TAS (1.67 ± 0.11 vs. 1.62 ± 0.12 mmol·L^-1^) (Figures 4, 5), there must be another biomarker no measured in this study leading to that higher muscle soreness observed in the *Cloudboom Echo 3* shoe model.

Despite the attenuated exercise-induced muscle damage responses and the lack of differences between conditions, there were significant biomechanical changes during the prolonged effort in this study. During the 60-min effort, participants tended to increase their SF (0.17%, 169.25 ± 2.15 vs. 169.54 ± 1.88 steps·min^-1^) and consequently reduce their SL (0.19%, 154.00 ± 3.00 vs. 153.70 ± 2.70 cm). Due fatigue, participants also experienced a significant increase in DF (0.71%, 28.00% ± 0.60% vs. 28.20% ± 0.50%) and a decrease in FT (1.06%, 156.61 ± 5.74 vs. 154.95 ± 5.14 ms), VO (1.20%, 9.19 ± 0.36 vs. 9.08 ± 0.32 cm) and ID (3.37%, 66.22 ± 20.52 vs. 64.02 ± 20.95 ms). These types of adaptations to fatigue have been previously reported and suggest that for constant speed during a prolonged run, trained athletes prefer to increase the number of steps and reduce their length, as it requires less intensity of vertical impacts (Giandolini et al., 2016; Morin et al., 2011). Although these fatigue-induced biomechanical adaptations, no differences were detected between footwear conditions and shoe × time interaction, indicating that these temporal adaptations likely reflect generalized fatigue responses rather than effects of footwear mechanic characteristics. This absence of condition effects may be partly explained by the limited sensitivity of the selected spatiotemporal and trunk accelerometry-derived variables to capture shoe-related differences. Furthermore, the 60-min duration, while sufficient to elicit moderate fatigue, might not replicate the biomechanical alterations expected during longer running protocols, such as half-marathon or marathon distances, where cumulative effects become more evident.

In this regard, Cartón-Llorente et al. (2022) found that, when trained athletes performed a 60 min time trial on a treadmill, no biomechanical adaptations occurred during the test. The reason why may be that participants were allowed to make variations on speed during the test. In longer efforts, such as a 24 h treadmill run, previous literature shows that participants tend to evolve to a running style which characterised by a higher step frequency and less vertical oscillation (Morin et al., 2011). These mechanisms have been also reported in ultra-marathon racing conditions (Giandolini et al., 2016), and seem to be protective adaptations to reduce the intensity of vertical impacts at each step (Patoz et al., 2023) and be more efficient for running economy (Folland et al., 2017). Although in this study, our test was a significantly shorter effort than an ultramarathon or a 24 h treadmill run, we found similar adaptations: the reduction in VO, FT, DF and ID, which imply that athletes made a transition to a more protective running style requiring less intensity of vertical impacts., and seem to be protective adaptations to reduce the intensity of vertical impacts at each step (Patoz et al., 2023) and be more efficient for running economy (Folland et al., 2017).

This study faced several limitations. These include the relatively small sample (*n* = 12) for some of the biomarkers, such as IL-6, and the fact that this study only tested men. This makes it difficult to generalise the results obtained as AFT might potentially have greater performance effects in women (Mason et al., 2024). Also, despite participants were asked to mimic their food, hydration and sleep patterns within the tests, no monitoring of these aspects was performed, which could have influenced the obtained results. Similarly, biomechanical parameters measured in this study might vary from overground running and motorized treadmill running (Riley et al., 2008). In addition, the outcome measures related to muscle damage, metabolic and oxidative stress are relatively basic, and it may be that other biomarkers related to inflammation and soreness (C-reactive protein, aspartate aminotransferase, myoglobin, troponin.) or oxidative stress (protein carbonyl, thiobarbituric acid reactive substances, total antioxidant capacity.), not measured here, are determinants of the DOMS differences observed between condition in this study. Also, the chosen timing for blood samples and DOMS assessment may have missed transient peaks in IL-6 and cortisol for blood samples, and 24–72 h peak dynamics for subjective measures. Future studies should include immediate and 2–6 h post-exercise blood samples and more timepoints for DOMS dynamics, as well as other objective metrics such as pressure pain threshold or creatine kinase correlation.

In conclusion, according to our results, both the *Cloudboom Strike* and the *Cloudboom Echo* 3 AFT shoe models appear to provide notable benefits with regard to reducing muscle damage, oxidative and metabolic stress, soreness, and inflammation in highly trained/national level -tier 3- male runners. Despite the lack of differences between conditions in the muscle damage, oxidative and metabolic stress biomarkers measured, the *Cloudboom Strike* implied significantly lower DOMS 24 h post-exercise than the *Cloudboom Echo* 3. These differences might be attributed to a biomarker that was not measured in this protocol. In addition, and in agreement with previous research, in both conditions, during the 60-min run, participants tended to increase their SF, reduce their SL, and, due to fatigue, also experienced a significant increase in DF and a decrease in FT, VO, and ID. However, there were no biomechanical differences between conditions.

## Data Availability

The raw data supporting the conclusions of this article will be made available by the authors, without undue reservation.
